# Development of the InTelligence And Machine LEarning (TAME) Toolkit for Introductory Data Science, Chemical-Biological Analyses, Predictive Modeling, and Database Mining for Environmental Health Research

**DOI:** 10.3389/ftox.2022.893924

**Published:** 2022-06-22

**Authors:** Kyle Roell, Lauren E. Koval, Rebecca Boyles, Grace Patlewicz, Caroline Ring, Cynthia V. Rider, Cavin Ward-Caviness, David M. Reif, Ilona Jaspers, Rebecca C. Fry, Julia E. Rager

**Affiliations:** ^1^ The Institute for Environmental Health Solutions, Gillings School of Global Public Health, The University of North Carolina at Chapel Hill, Chapel Hill, NC, United States; ^2^ Department of Environmental Sciences and Engineering, Gillings School of Global Public Health, The University of North Carolina at Chapel Hill, Chapel Hill, NC, United States; ^3^ Research Computing, RTI International, Durham, NC, United States; ^4^ Center for Computational Toxicology and Exposure, US Environmental Protection Agency, Durham, NC, United States; ^5^ Division of the National Toxicology Program, National Institute of Environmental Health Sciences, Durham, NC, United States; ^6^ Center for Public Health and Environmental Assessment, US Environmental Protection Agency, Chapel Hill, NC, United States; ^7^ Bioinformatics Research Center, Department of Biological Sciences, North Carolina State University, Raleigh, NC, United States; ^8^ Curriculum in Toxicology and Environmental Medicine, School of Medicine, University of North Carolina, Chapel Hill, NC, United States; ^9^ Center for Environmental Medicine, Asthma and Lung Biology, School of Medicine, University of North Carolina, Chapel Hill, NC, United States; ^10^ Department of Pediatrics, Microbiology and Immunology, School of Medicine, University of North Carolina, Chapel Hill, NC, United States

**Keywords:** bioinformatics and computational biology, cheminformatics, data science, epidemiology, exposure science, machine learning, public health, toxicology

## Abstract

Research in environmental health is becoming increasingly reliant upon data science and computational methods that can more efficiently extract information from complex datasets. Data science and computational methods can be leveraged to better identify relationships between exposures to stressors in the environment and human disease outcomes, representing critical information needed to protect and improve global public health. Still, there remains a critical gap surrounding the training of researchers on these *in silico* methods. We aimed to address this gap by developing the inTelligence And Machine lEarning (TAME) Toolkit, promoting trainee-driven data generation, management, and analysis methods to “TAME” data in environmental health studies. Training modules were developed to provide applications-driven examples of data organization and analysis methods that can be used to address environmental health questions. Target audiences for these modules include students, post-baccalaureate and post-doctorate trainees, and professionals that are interested in expanding their skillset to include recent advances in data analysis methods relevant to environmental health, toxicology, exposure science, epidemiology, and bioinformatics/cheminformatics. Modules were developed by study coauthors using annotated script and were organized into three chapters within a GitHub Bookdown site. The first chapter of modules focuses on introductory data science, which includes the following topics: setting up R/RStudio and coding in the R environment; data organization basics; finding and visualizing data trends; high-dimensional data visualizations; and Findability, Accessibility, Interoperability, and Reusability (FAIR) data management practices. The second chapter of modules incorporates chemical-biological analyses and predictive modeling, spanning the following methods: dose-response modeling; machine learning and predictive modeling; mixtures analyses; -omics analyses; toxicokinetic modeling; and read-across toxicity predictions. The last chapter of modules was organized to provide examples on environmental health database mining and integration, including chemical exposure, health outcome, and environmental justice indicators. Training modules and associated data are publicly available online (https://uncsrp.github.io/Data-Analysis-Training-Modules/). Together, this resource provides unique opportunities to obtain introductory-level training on current data analysis methods applicable to 21st century science and environmental health.

## Graphical Abstract

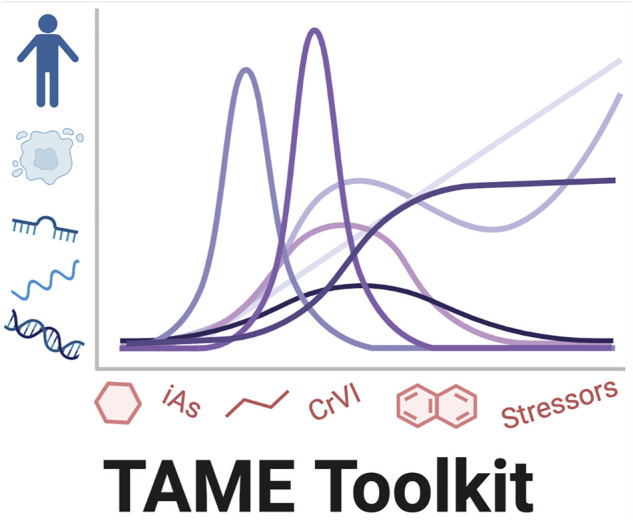

## Highlights

• Training that translates data science into environmental health research is needed
• Modules were developed to teach coding basics and introductory data science
• Also cover chemical-biological modeling, machine learning, and database mining
• Modules exemplify methods to uniquely address environmental health issues
• Modules allow for improved training towards current data analysis methods

## 1 Introduction

The field of environmental health is rapidly expanding efforts aimed at the improved data science methods and data integration. Data produced in environmental health studies are becoming larger, with increased resolution and expanded variable coverage paralleling technological advancements and improved record keeping. These data now serve as critical resources to increase the understanding of relationships between chemicals in the environment and disease outcomes. Multiple organizations have recently advocated for increased reliance and proficiency surrounding *in silico* approaches to advance the science of toxicity testing, improve chemical exposure assessments, and increase data sharing and associated analysis tools (NAS, 2007; NAS, 2017; EU, 2019; Florance, 2020; Sim et al., 2020; EPA, U.S, 2021b). However, there remains high demand for personnel that are adequately trained to analyze and manage large datasets to address environmental health issues, representing a timely concern that requires updated resources and training opportunities. We therefore aimed to contribute towards this critical gap through the development of an online toolkit, titled the inTelligence And Machine lEarning (TAME) Toolkit, to promote didactic data generation, management, and analysis methods to “TAME” data in environmental health studies.

The TAME Toolkit was developed to provide a publicly available, self-guided tour on topics spanning introduction to computer programming, chemical-biological analyses, predictive modeling, and environmental health database mining. The majority of computer programming information and examples provided within the TAME Toolkit were based in the R coding language, since this coding environment is publicly available, widely used, and well-documented (The R Project for Statistical Computing, 2021). R is specifically available as Free Software under the Free Software Foundation’s GNU General Public License and can be run across all major platforms and operating systems, including Unix, Windows, and MacOS. Because of this open licensing format, R has emerged as an avenue for world-wide collaboration, benefiting from the continual expansion through thousands of user-developed packages that aid in improved data analyses and methods sharing. Packages have varying utilities, spanning basic organization and manipulation of data to cutting-edge approaches to parse and analyze data through artificial intelligence (AI) and/or machine learning (ML) (CRAN, 2021a; Bioconductor, 2021).

Data analysis examples were included in the TAME Toolkit to span topics relevant to environmental health, which is notably multi-disciplinary and includes exposure science, epidemiology, toxicology, bioinformatics/cheminformatics, and related disciplines. Examples were developed by the team of authors, pulling from their real-world datasets and expertise in environmental health data analytics. Training modules were organized to include examples of each authors’ area of expertise, to provide a broad foundation in data science methods relevant to environmental health. Modules contained within the TAME Toolkit were organized into three chapters spanning 1) introductory data science; 2) chemical-biological analyses and predictive modeling; and 3) environmental health database mining. Modules were designed to aid in the training of students, post-baccalaureate and post-doctorate trainees, and professionals that are interested in expanding their skillsets surrounding data analysis techniques relevant to environmental health, toxicology, exposure science, epidemiology, and bioinformatics/cheminformatics. These modules will continue to be expanded and improved upon in the coming years, to continue the expanded use of data management and analysis tools to address timely environmental health research topics and promote meaningful collaborations across this multi-disciplinary field of study.

## 2 Methods

### 2.1 Overall Approach to Organizing the inTelligence And Machine lEarning Toolkit

**FIGURE 1:**
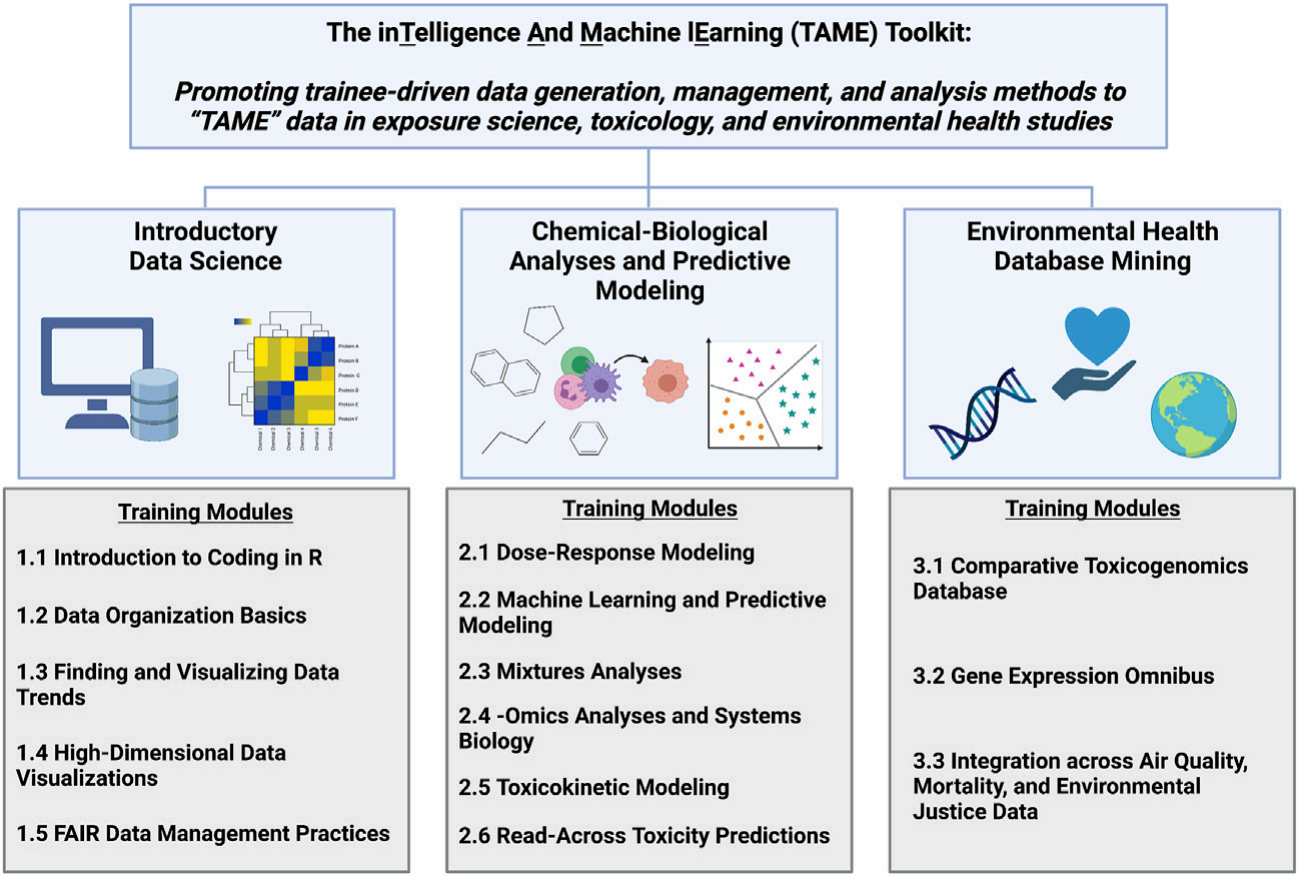
Overall organization of the TAME Toolkit, developed to promote trainee-driven data generation, management, and analysis methods to “TAME” data in exposure science, toxicology, and environmental health research. Individual training modules were developed in R coding language to provide applications-based training in the broad categories of data science, chemical-biological analyses and predictive modeling, and environmental health database mining.

The TAME Toolkit was developed with the goal of guiding participants with various backgrounds through data organization and analysis methods that are useful towards evaluating big data in exposure science, epidemiology, toxicology, and environmental health studies. Modules were developed to cover three primary focuses (organized into chapters): 1) introductory data science; 2) chemical-biological analyses and predictive modeling; and 3) environmental health database mining (Figure 1). Applications-based environmental health questions are posed to keep participants actively engaged. These questions also aid in the translation of methods towards real-world exposure science, toxicology, and public health issues. These modules were developed based on examples from our ongoing research efforts using environmental health datasets and/or related data generated for these training purposes.

The TAME Toolkit was developed to provide an overview of example approaches to analyzing data that are highly relevant to exposure science, toxicology, and environmental health research applications. Rather than including an exhaustive list of techniques and tutorials that covers all potentially relevant methods, we instead highlight example methods and datasets that are tangible and cover important aspects of data organization, visualization, and analysis within the environmental health research field. Topics of training modules were selected to include current approaches that are of high interest in 21st century toxicology and exposure science and new approach methodologies (NAS, 2007; NAS, 2017; Wambaugh et al., 2019; Zavala et al., 2020) that also align with the coauthors’ areas of expertise. These examples are provided in the TAME Toolkit through organized training modules that are purposely stand-alone and discrete, as opposed to organizing modules that depend on participants having successfully completed all preceding modules and associated analyses. This format was selected to allow participants to engage in specific analysis topics they are interested in learning in achievable spans of time.

Modules were developed to include helpful resources throughout the code, such that users aiming to further their education/development have access to additional learning opportunities and analysis methodologies to explore. Notably, each module was not designed as a complete guide to conduct research on a specific topic; rather, modules were designed as a starting point for a data analysis technique. Additional guidance and resources were incorporated throughout each of the training modules, particularly within the introduction sections as well as the final concluding remarks sections, to point participants to additional examples and guidance when interested. These additional resources spanned book chapters and guidance documents dedicated to the specific data analysis topic, as well as example peer-reviewed, published literature. All concepts within the TAME Toolkit were selected to include important techniques that can be incorporated into environmental health studies, and thus, provide a cohesive set of skills that trainees can leverage within their current research studies, real-world applications, and/or future job marketability, depending on their career stage.

### 2.2 Target Audience of the TAME Toolkit

Data training modules were designed for the following target audiences: 1) academic students obtaining degrees in environmental health, toxicology, exposure science, epidemiology, bioinformatics, and related disciplines; 2) post-baccalaureate and post-doctorate trainees that are working in the environmental health research arena; 3) professionals in academia, government, or industry that are interested in expanding their skillset to include recent advances in data analysis methods relevant to environmental health, toxicology, exposure science, epidemiology, and bioinformatics/cheminformatics. These participants would ideally have some level of training in basic biology, chemistry, environmental science, toxicology, and/or epidemiology, though training modules were organized to provide background information and helpful resources to provide background reading/training materials for content that participants may benefit from if lacking a certain background across the multi-disciplinary field of environmental health.

### 2.3 Data Training Module Development and Underlying Technologies

The TAME training modules were developed and made publicly available through the UNC Superfund Research Program (UNC-SRP) Github website, specifically through a Bookdown website available at: https://uncsrp.github.io/Data-Analysis-Training-Modules/. This interface was selected as the primary landing site for these modules because of its smooth communication between R/RStudio/Markdown. Furthermore, all module example datasets and script files could be easily organized and posted to the parent Github webpage, publicly available at: https://github.com/UNCSRP. Github was selected as the primary warehouse of these data and associated script, as it currently represents the most commonly used platform for storing, tracking, and collaborating on software/computing projects spanning over 73 million developers, 4 million organizations, and 200 million repositories (Github, 2022). We organized the TAME Toolkit script and underlying data to be available to participants through both Bookdown and Github to meet the learning preferences of each participant. This structure specifically allows participants to follow through finalized training modules online that are published through Bookdown, and it also allows participants to download the raw data and script from the parent Github webpage and run the modules on their own computers through their local computing systems and preferred programming structure.

Each training module was specifically developed in R Markdown, which is a LaTeX-like documentation format that allows developers to draft comprehensive documentation throughout R-based scripts (Baumer and Udwin, 2015). R Markdown is also advantageous in that programmers can run their code in entirety on their computer and save a “knitted” version of the code that also displays messages, results, and graphics that are produced when running each line of code. These R Markdown files (with .Rmd extensions) represent the specific script documents that were uploaded to Github, alongside README files and associated input/output data files used during the scripted activities. The final knitted html files of each training module’s R Markdown script represent the actual file that was used in the online posting to Bookdown.

### 2.4 Data Training Module Evaluation

Modules were beta tested through the delivery of teaching materials within Dr. Julia Rager’s new course at UNC, titled “Computational Toxicology and Exposure Science.” UNC students, largely consisting of graduate-level students in environmental science, toxicology, and public health, provided feedback on each module’s content *via* anonymous course surveys and classroom-led conversations. These suggestions were incorporated into the final versions of the training modules with the goal of achieving the TAME Toolkit objectives and broadening target audiences and dissemination into the greater scientific community. Select training modules have also been disseminated *via* hands-on training workshops. Feedback surrounding training module content was similarly collected *via* anonymous course surveys and incorporated into revised training materials.

### 2.5 TAME Toolkit Contributors

TAME Toolkit content and associated training modules were developed by experts in environmental health research and data science. These experts were selected to contribute to the TAME Toolkit based on the following qualifications: Dr. Kyle Roell is the lead Data Analyst for the Institute for Environmental Health Solutions at the University of North Carolina at Chapel Hill (UNC) and is an experienced software developer and programmer with expertise in bioinformatics and statistical genetics (Zhang et al., 2017; Roell et al., 2019; Roell et al., 2021). Ms. Koval is a graduate student in the UNC Department of Environmental Sciences and Engineering and is contributing towards ongoing studies on human health and the environment (Ring et al., 2021; Carberry et al., 2022). Rebecca Boyles is a Director of the Center for Data Modernization Solutions at RTI International. Ms. Boyles is an expert at data driven research collaborations and the implementation of computational approaches to ensure research data are Findable, Accessible, Interoperable, and Reusable (FAIR) (Boyles et al., 2019; Robasky et al., 2020; Holmgren et al., 2021). Dr. Grace Patlewicz is a Chemist at the U.S. Environmental Protection Agency (U.S. EPA) and leader of chemical read-across applications towards chemical safety assessments (Helman et al., 2019b; Nelms et al., 2020; Shah et al., 2021). Dr. Caroline Ring is a Principal Investigator at the U.S. EPA and leader in computational exposure science and toxicology approaches for chemical regulatory safety assessments, with particular expertise in toxicokinetics (Ring et al., 2017; Ring et al., 2019; Ring et al., 2021). Dr. Cynthia Rider is a Toxicologist at the National Institute of Environmental Health Sciences and a leading expert in the chemical safety and risk assessment of chemical mixtures (Catlin et al., 2018; Ryan et al., 2019; Rider et al., 2021). Dr. Cavin Ward-Caviness is a Computational Biologist and Principal Investigator at the U.S. EPA, and he leads studies integrating geospatial exposure measures with molecular biomarkers and health outcome data to understand the impacts of chemical pollutants and social determinants of health (Ward-Caviness et al., 2020; Martin et al., 2021; Ward-Caviness et al., 2021). Dr. David Reif is a Professor in the Department of Biological Sciences at North Carolina State University (NCSU) and Director of the NCSU Bioinformatics Consulting and Services Core. Dr. Reif leads studies implementing computational modeling approaches to leverage big data in predicting exposure and disease outcomes (Kosnik and Reif, 2019; Green et al., 2021; Marvel et al., 2021). Dr. Jaspers is a Professor in the Department of Pediatrics, Microbiology and Immunology at UNC, and is the Director of the Curriculum in Toxicology and Environmental Medicine and Director of the Center for Center for Environmental Medicine, Asthma and Lung Biology. Dr. Jaspers leads studies integrating medicine with environmental health research, combining data from clinical, toxicological, and molecular biology study designs (Jaspers et al., 1997; Rager et al., 2013; Rebuli et al., 2021). Dr. Fry is a Professor of Environmental Sciences and Engineering and is the Director of the UNC-Chapel Hill Superfund Research Program and the Director of the Institute for Environmental Health Solutions. Dr. Fry leads studies integrating genomic and epigenomic approaches within epidemiological, toxicological, and clinical study designs to identify mechanisms of environmental exposure-induced disease and organize solution-oriented intervention (Fry et al., 2007; Smeester et al., 2011; Fry et al., 2012; Manuck et al., 2021a). Dr. Julia Rager is an Assistant Professor in the UNC Department of Environmental Sciences and Engineering, and she leads studies evaluating the health impacts of environmental exposures through bioinformatic approaches aimed at integrating chemical-biological signatures to elucidate primary disease drivers and their underlying biological mechanisms (Rager et al., 2015; Rager et al., 2017; Clark et al., 2021; Rager et al., 2021). Collectively, this team of environmental health research experts were well-equipped to develop training materials within the TAME Toolkit.

## 3 Results

TAME Toolkit training modules are now publicly available, promoting trainee-driven data generation, management, and analysis methods to “TAME” data in environmental health studies. These modules are publicly accessible (https://uncsrp.github.io/Data-Analysis-Training-Modules/), with underlying code and datasets available in the parent UNC-SRP GitHub website (https://github.com/UNCSRP). Descriptions of each training module are provided below alongside their associated datasets and primary analysis findings. Collectively, training modules serve as representative examples that address research questions relevant to environmental health including topics of toxicology, exposure science, epidemiology, bioinformatics, and related fields of study.

### 3.1 Introductory Data Science

This series of TAME Toolkit training modules begins with introductory-level training on setting up R/RStudio, coding, data organization basics, basic methods to identify and visualize trends in data, and visualize high-dimensional data (modules 1.1–1.4). Introductory data science materials have previously been covered by other groups/online resources (Wickham and Grolemund, 2017; Adair et al., 2021; Coursera, 2021), and we therefore provide a high-level overview of these introductory modules below. A more focused description begins with the next training module, which serves as a novel introduction to FAIR data management practices (module 1.5). This module is also the first to incorporate questions specific to environmental health, which are included throughout the remaining training modules.

#### 3.1.1 Introduction to Coding in R

The objective of this module is to provide an introduction to coding through the R language and its associated environment, RStudio. This objective is met by first detailing instructions with corresponding screenshots describing how to download/install both of these programs. An introduction on installing and loading packages in R is then provided. Scripting basics are detailed, including setting a working directory, importing and exporting files, and viewing data within the R console/RStudio environment. The importance of this module is that it provides the foundation needed for participants to become acclimated and set-up for running R programming on their computing systems.

#### 3.1.2 Data Organization Basics

The objective of this module is to provide an introduction on data organization methods. This objective is met by presenting basic data organization methods using an example environmentally relevant human cohort dataset. This cohort was generated by creating data distributions randomly pulled from our previous publications (Rager et al., 2014a; Clark et al., 2019; Payton et al., 2020; Clark et al., 2021), resulting in a bespoke dataset for these training purposes. Data include subject information/demographic data, as well as environmental exposure data, focusing on metals concentrations in drinking water and human urine samples. Data organization methods that are demonstrated in this training module include merging, filtering, subsetting, melting, and casting. These important methods are demonstrated using base R functions, as well as the commonly implemented package, *Tidyverse*, that allows users to more efficiently organize and manipulate datasets in R (CRAN, 2021b). The importance of this module is that it provides basic skills needed to organize data, in general, within the coding environment, representing a foundational skill that must be acquired prior to running any scripted analysis.

#### 3.1.3 Finding and Visualizing Data Trends

The objective of this module is to provide an overview of basic statistical tests and data visualizations. This objective is met leveraging the same example cohort with environmentally relevant data introduced in Section 3.1.2. Tests for normality are first presented, alongside methods to plot histograms and boxplots to view data distributions. Basic statistical tests are then presented, including the *t*-test, analysis of variance, regression modeling, chi-squared test, and Fisher’s exact test. Additional example visualizations are provided alongside these statistical tests, including boxplots, scatterplots, and regression lines. These statistical tests are introductory-level, with more extensive examples and associated descriptions of statistical models in the proceeding applications-based training modules (e.g., modules 2.4, 2.5, 2.6, 3.2, and 3.3). The importance of this module is that it provides an overview of statistical methods that are very routinely employed within environmental health studies, and thus learning how to carry out these basic statistics represents a foundational skillset for anyone in this field of study.

#### 3.1.4 High-Dimensional Data Visualizations

The objective of this module is to provide an introduction to methods that can be used to visualize high dimensional data. Approaches described in this training module include data formatting, data scaling, and the visualization of prepared datasets through density plots, GGally plots, boxplots, correlation plots, hierarchical clustering, and heatmaps. Visualization approaches are demonstrated using a large environmental chemistry dataset, based off a chemical analysis of smoke samples collected during lab-based simulations of wildfire events. These data have been previously published (Kim et al., 2018; Rager et al., 2021) and are used here as an example of an environmental dataset relevant to environmental health. These visualization methods are provided here at an introductory-level, with many other examples detailed throughout the majority of the next training modules. The importance of this module is that it provides ideas and techniques that can be used to visualize data relevant to environmental health, which are becoming increasingly high dimensional and thus, require these more sophisticated methods to adequately illustrate important data trends.

#### 3.1.5 Findability, Accessibility, Interoperability, and Reusability Data Management Practices

The objective of this module is to introduce trainees to best data management practices in environmental health research. A method to ensure proper data management is the implementation of Findability, Accessibility, Interoperability, and Reusability (FAIR) practices (Wilkinson et al., 2016). This topic is receiving much attention in recent years through workshops, government reports, and publications which are published within the online training module. The following questions are addressed throughout this training module:

1) What is FAIR?
2) When was FAIR first developed?
3) When making data “Findable,” who and what should be able to find your data?
4) When saving/formatting your data, which of the following formats is preferred to meet FAIR principles: .pdf, .csv, or a proprietary output file from your instrument?
5) How can I find a suitable data repository for my data?

**FIGURE 2:**
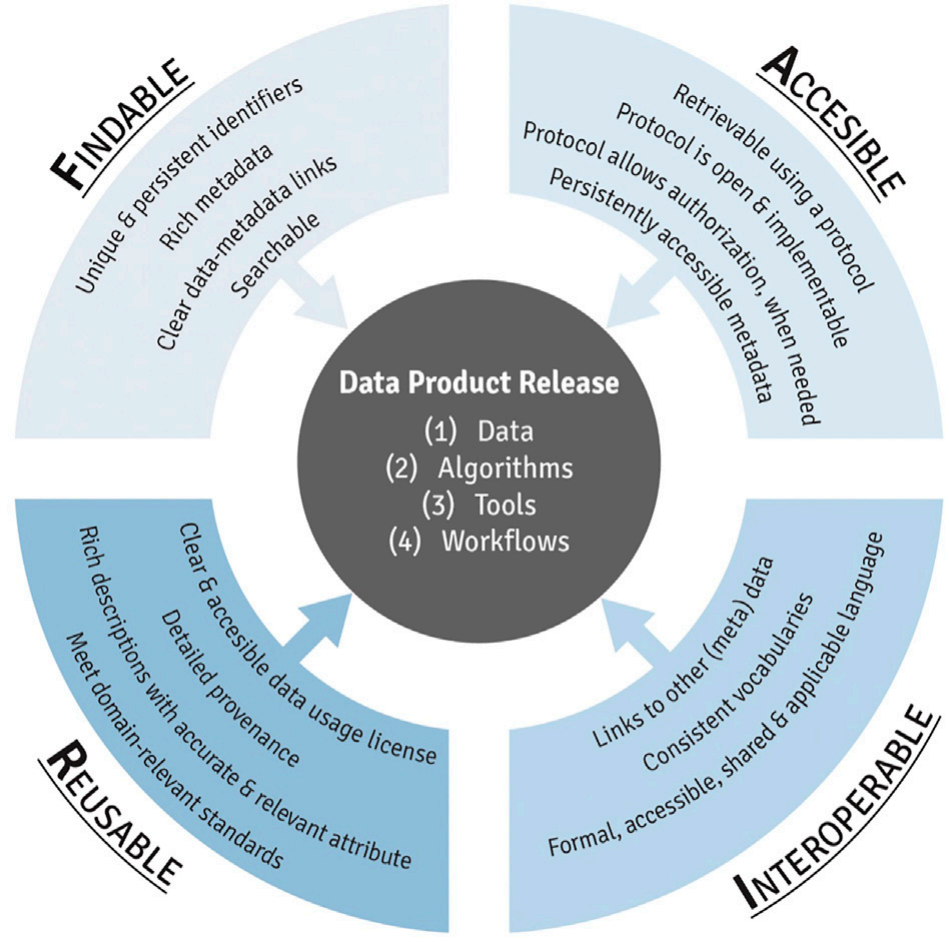
An overview of the individual components of FAIR data management practices, resulting in the effective release of data products from exposure science, epidemiology, toxicology, and environmental health research.

This module first provides an introduction to FAIR (Figure 2), including a history of how this term was first developed and implemented. Trainees are then guided through each component of FAIR, organized by letter. To detail, the F in FAIR identifies components needed to make the meta(data) findable. These components include unique persistent identifiers and descriptive information (i.e., metadata) that can be searched by both humans and computer systems. The A components are designed to enable that meta(data) be available long-term, and accessed by humans and machines using standard communication protocols with clearly described limitations on reuse. The I components of the principles address needs for data exchange and interpretation by humans and machines which includes the use of controlled vocabularies or ontologies to describe meta(data) and to describe provenance relationships through appropriate data citation. The R components highlight needs for meta(data) to be reused and support integration such as sufficient description of the data and data use limitations. The training module then reviews different types of data repositories that can be used to publish datasets in exposure science, toxicology, and environmental health research. Lastly, this module provides participants with additional training resources, workshops, government reports, and example publications surrounding the use of FAIR data management practices. The importance of this module is that effective data management, organization, and longevity are becoming increasingly critical in ensuring studies are scientifically sound and reproducible, and thus, all scientists that are involved in the analysis of data for a project should be aware of these issues and implement them within their ongoing studies. Research funding agencies are additionally requiring increased attention surrounding data sharing and FAIR practices (NIH, 2022).

### 3.2 Chemical-Biological Analyses and Predictive Modeling

This chapter of TAME Toolkit training modules covers approaches that can be used to carry out chemical and/or biological analyses and predictive modeling to better understand exposure-induced disease and underlying toxicological mechanisms. Modules span topics of dose-response modeling (module 2.1), machine learning and predictive modeling (2.2), mixtures analyses (2.3), -omics analyses and systems biology (2.4), toxicokinetic modeling (2.5), and read-across toxicity predictions (2.6). Environmental health questions are posed throughout these modules to maintain active engagement and provide tangibility on the use of the described methods towards environmental health applications. These training modules are detailed below.

#### 3.2.1 Dose-Response Modeling

The objective of this module is to provide an overview on analyzing toxicological response data in relation to exposure concentrations (or doses), resulting in the derivation of benchmark doses (BMDs). This topic is of high relevance to the field of environmental health, as BMDs represent values that are commonly used as the basis for evaluating risk in chemical safety evaluations, informing the levels at which chemicals may be regulated. This module specifically analyzes animal tumor incidence rates in response to exposure to a mock chemical tested across 12 different concentrations in drinking water. This dataset was generated for the specific purposes of this exercise, to allow for some interesting curve fits and a comparison between tissue site sensitivity to an example chemical insult. Several environmental health questions are posed throughout this module, including:

1) Which target tissue demonstrated the highest incidence of tumor formation from any single exposure dose?
2) Which target tissue’s tumor incidence seems to not be related to dose?
3) Upon visual inspection of example log-logistic vs. Weibull model curve fits, can we confidently determine which of these two models best fits these data?
4) For the liver tumor response data, which model curve fits the resulting dose-response data the best? What are the final resulting BMD and BMDL estimates from this model?
5) In comparing between the intestinal vs. liver datasets, which tissue is estimated to show tumor responses at a lower exposure dose?

**FIGURE 3:**
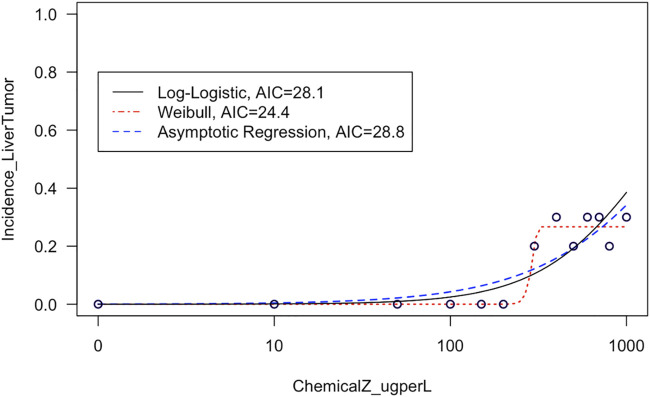
Example model curves that are fit to liver tumor incidence data in this training module. This training module guides trainees through the plotting of dose-response data and the fitting of different types of models to describe dose-response trends in these data. The fit of each resulting curve is evaluated through visual inspection and evaluation of AIC values, and then the training module focuses on the model with the lowest AIC to derive benchmark dose estimates.

This module first provides a high-level introduction to BMD modeling, and then guides trainees through the process of downloading/loading required packages and example data used in this exercise. These data are then viewed, such that trainees can see the four different tissue sites evaluated for carcinogenicity in response to exposure (i.e., kidney, liver, intestinal, and stomach tissues) and also obtain information on the overall distributions of tissue-specific tumor incidence. Then, data are plotted in dose-response using standard scatter plots with exposure concentrations along the *x*-axis and tumor incidence along the *y*-axis. With these foundation plots generated, trainees are then guided through methods to fit various model curves to these dose-response data, spanning log-logistic, Weibull, and asymptotic regression models as core examples available through the *drc* package (Ritz et al., 2015). The best fitting curves are then identified through 1) visual inspection of curve fits, and 2) calculation of Akaike Information Criterion (AIC) values. These examples highlight the importance of evaluating model fit to ultimately determine which model should be used to derive final BMD estimates (Figure 3). Trainees are lastly pointed to example dose-response publications that have addressed environmental health questions (Rager et al., 2017; Auerbach and Paules, 2018; Thompson et al., 2018; Johnson et al., 2020), as well as additional modeling tools and guidance documents surrounding dose-response assessments. The importance of this module is that BMD modeling represents a foundational topic in environmental health, where methods can be used to better understand which exposure concentrations/doses are required to elicit toxicity by evaluating trends in datasets.

#### 3.2.2 Machine Learning and Predictive Modeling

The objective of this module is to provide an overview of machine learning (ML) approaches to evaluate high dimensional data relevant to environmental health applications. This module begins by introducing the need for predictive modeling, defining its use in the context of toxicology and environmental health, then establishing a working distinction between ML and traditional statistical methods. Recognizing the wide variety of machine learning methods currently available to researchers, this training module presents introductory-level information on two commonly employed methods, principal component analysis (PCA) and k-means clustering. Their use is illustrated on real data obtained from the National Toxicology Program’s Integrated Chemical Environment (ICE) resource (https://ice.ntp.niehs.nih.gov/). This module analyzes an example dataset of physicochemical property information for chemicals spanning two classes: per- and polyfluoroalkyl substances (PFAS) and statins. PFAS represent a ubiquitous and pervasive class of man-made industrial chemicals of high environmental relevance due to their persistence in the environment after contamination events (Fenton et al., 2021). Statins represent a class of lipid-lowering pharmaceuticals used for patients at risk of cardiovascular disease, and statins have been identified as present in water/wastewater effluent (Tete et al., 2020). The applied data example in this training module was designed to illustrate the concept of using ML methods to differentiate chemical class and “predict” (in this case, the group membership is known) chemical groupings that can inform a variety of environmental and toxicological applications. The following environmental health questions are addressed throughout this training module:

1) Can we differentiate between PFAS and statin chemical classes, when considering just the raw physicochemical property variables without applying machine learning techniques?
2) What are some of the physicochemical properties that seem to be driving chemical clustering patterns derived through k-means?
3) Upon reducing the data dimensionality through PCA, which physicochemical property contributes the most towards informing data variance captured in the primary principal component?
4) How do the data compare when physicochemical properties are reduced using PCA?
5) If we did not have information telling us which chemical belonged to which class, could we use PCA and k-means to accurately predict whether a chemical is a PFAS vs. statin?
6) What kinds of applications/endpoints can be better understood and/or predicted, because of these derived chemical groupings?

**FIGURE 4:**
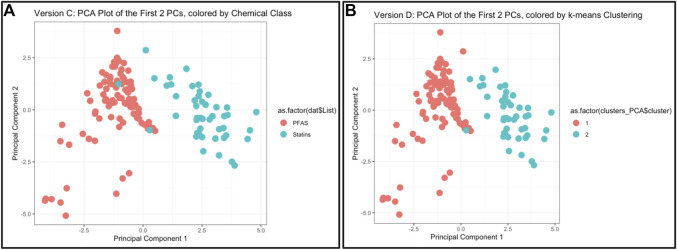
Example results and visualizations produced through this machine learning and predictive modeling activity, based on an example dataset of 144 PFAS and statins. **(A)** Principal components were derived to capture the majority of variance amongst physicochemical properties across PFAS and statins, using PCA methods. **(B)** Chemical classes were predicted using k-means clustering across PCA-reduced components, demonstrating that the predicted chemical classes were almost identical to the actual chemical classes [as shown in **(A)**].

This module first provides a high-level introduction to the topics of machine learning and predictive modeling, and then guides trainees through the process of downloading/loading required packages and example data used in this exercise. These data are then viewed by plotting chemicals along their native physicochemical scales (e.g., boiling point versus molecular weight) for all 144 different chemicals, colored according to the two classes of PFAS and statins. Visualizing these data through two bivariate plots demonstrates that there is signal in the data, but substantial overlap between classes for most properties, i.e., individual physicochemical properties may not clearly differentiate between chemical classes. This limitation substantiates the need to employ machine learning methods to better describe group-level trends in these data. K-means clustering is then performed across all physicochemical property data in their native scale and visualized using a heat map. Next, PCA is carried out across all physicochemical property data and visualized to illustrate the concept of dimensionality reduction. The code is provided to calculate results from this PCA, including eigenvalues, percent variance captured by each principal component, and loading scores for the input variables. Finally, PCA is combined with k-means to generate predictions of two chemical groupings that almost entirely capture real-world classifications (Figure 4). Lastly, these methods are discussed in relation to additional applications, including the evaluation of other outcomes such as environmental fate and transport and disease outcome predictions. Trainees are provided additional resources including recent example studies that incorporate machine learning to address environmental health questions (To et al., 2019; Clark et al., 2021; Green et al., 2021; Odenkirk et al., 2021; Ring et al., 2021). The importance of this module is that it provides a helpful introduction to foundational ML concepts, and upon receiving this training, participants should be positioned to apply these methods to make predictions within their own high dimensional datasets.

#### 3.2.3 Mixtures Analyses

The objective of this module is to provide an overview of chemical composition signatures and toxicological responses that can be evaluated to inform whether complex mixtures are “sufficiently similar.” Results from these analyses, referred to as sufficient similarity analyses, can be used to inform data extrapolation from a data-rich mixture to a data-poor mixture during a chemical safety/risk assessment, to adequately protect human health. In this example, data are re-analyzed from a study evaluating the chemical composition and toxicological effects of *Ginkgo biloba* extract, a common dietary supplement ingredient that is commercially available in the U.S. (Catlin et al., 2018). Here, 29 different sample lots of *G. biloba* extract were collected from several suppliers and analyzed. The chemical components of these sample extracts were evaluated using targeted methods, and associated toxicity was evaluated using gene-specific *in vitro* response assays. These data are leveraged in this training module to inform which of the *G. biloba* samples are sufficiently similar (and thus could use the same toxicological data for risk evaluation), and which are different (and thus would require additional testing). Several questions are posed throughout this module, including:

1) When viewing the variability between chemical profiles, how many groupings of potentially “sufficiently similar” *G. biloba* samples do you see?
2) Which chemicals do you think are important in differentiating between the different *G. biloba* samples?
3) When viewing the variability between toxicity profiles, how many groupings of potentially “sufficiently similar” *G. biloba* samples do you see?
4) Were similar chemical groups identified when looking at just the chemistry vs. just the toxicity? How could this impact regulatory decisions?

**FIGURE 5:**
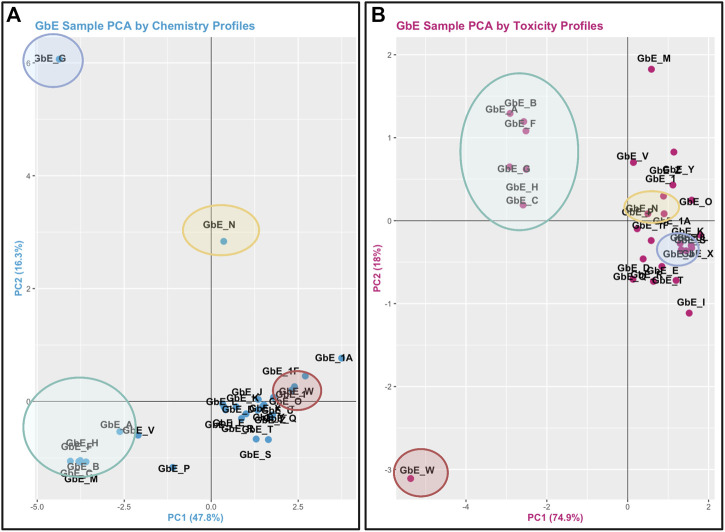
PCA plots used to inform Ginkgo Biloba extract (GbE) groupings in mixtures-based sufficient similarity analyses. **(A)** This training module first guides participants through the derivation of chemical groups within GbE produced when reviewing only chemical composition data. **(B)** Then, chemical groups are derived by reviewing *in vitro* toxicity response profiles associated with GbE exposures. These grouping results are then compared to highlight that important patterns may be missed when evaluating just chemistry or just toxicity response profiles in a mixtures-based sufficient similarity analysis. Groups are derived across these examples using PCA, representing a very common data reduction/visualization method used to explain the variance across high dimensional datasets.

This module specifically guides trainees through the loading of required packages and data, and then carries out an example sufficient similarity analysis first using the chemistry data. Trainees are guided through data processing and scaling, leading to two different grouping and visualization approaches: 1) PCA and associated scatter plot, and 2) hierarchical clustering and associated heat map visualization. Results are used to inform which *G. biloba* extracts display similar chemical composition profiles and which do not. Participants are also guided through the evaluation of potential outlier samples, gauging whether these impact overall data distributions. Similar methods are then used to evaluate the toxicological response data. This analysis concludes with a side-by-side comparison of the sample groupings that result when considering chemical composition vs. toxicity profile data (Figure 5), highlighting the importance of considering both data streams when determining sufficient similarity in the evaluation of complex mixtures. Trainees are then provided additional resources and references for further information on sufficient similarity analyses in environmental health research (Rice et al., 2009; Catlin et al., 2018; Ryan et al., 2019; Collins et al., 2020). The importance of this module stems from real-world exposures to complex mixtures that often have incomplete toxicity data, which necessitate training to determine when data from a reference mixture can be extrapolated to a mixture-of-concern for risk evaluation.

#### 3.2.4 -Omics Analyses and Systems Biology

The objective of this module is to provide an overview of the -omics field and its relation to environmental health research, highlighting transcriptomics as an important -omics endpoint to analyze as a scripted example. The field of -omics initiated from genome-wide information obtained through the Human Genome Project, and has since then expanded to include -omic endpoints spanning the genome, epigenome, transcriptome, proteome, metabolome, microbiome, and the exposome (Cho and Blaser, 2012; Wild, 2012; Rager and Fry, 2013; Clark and Rager, 2020). Stressors within the environment have the potential to alter -omic signatures, impacting downstream biological processes, cellular function, tissue phenotypes, and overall health (Rager and Fry, 2013; Clark and Rager, 2020; Rager et al., 2020). When interpreting the potential consequences of -omic alterations, it is often helpful to place findings into the context of systems biology. In these systems-level analyses, molecules can be overlaid onto molecular networks to uncover biological pathways and cellular functions that are altered under the condition being tested (Rager and Fry, 2013; Meisner and Reif, 2015). This training module provides an overview of these strategies, using an example transcriptomics dataset acquired from lung tissues of mice exposed to biomass burn conditions indicative of the potential wildfire exposure scenarios (Kim et al., 2018; Rager et al., 2021). Several questions are posed through this module, including:

1) What two input data files are commonly needed in the analysis of -omics (e.g., transcriptomics) data?
2) When preparing transcriptomics data for statistical analyses, what are common data filtering steps that are completed during the data QA/QC process?
3) How many genes showed significant differential expression in the mouse lung associated with flaming pine needles, smoldering pine needles, and lipopolysaccharide (LPS)?
4) What biological pathways are disrupted in association with flaming/smoldering pine needles exposure in the lung, identified through systems level analyses?

**FIGURE 6:**
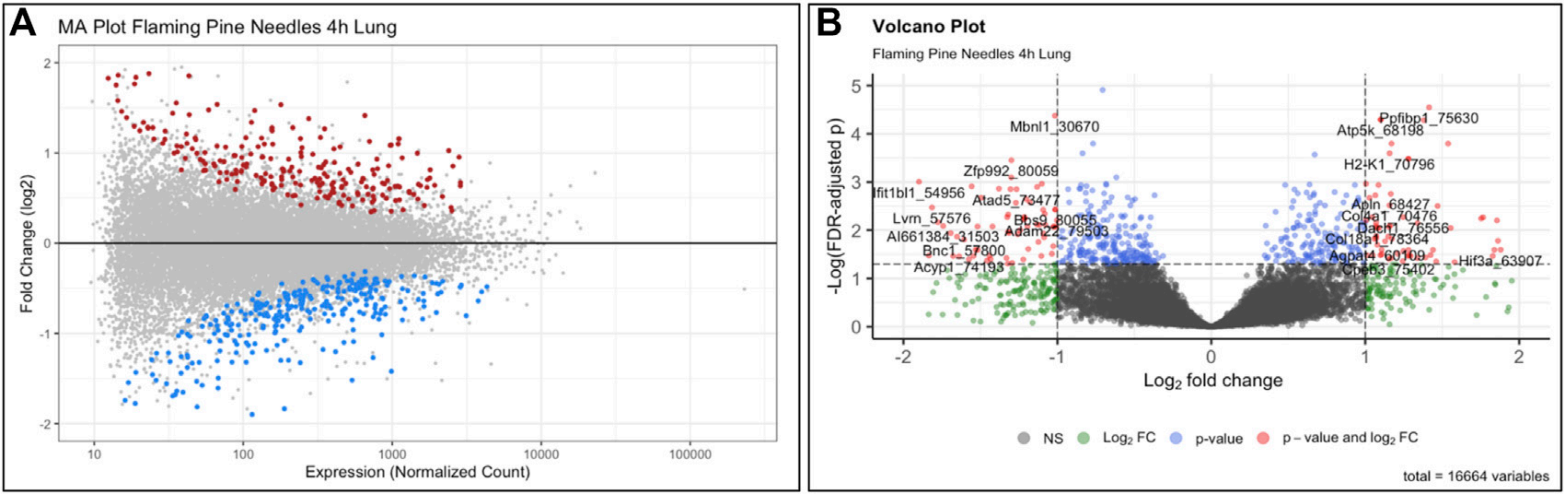
Example **(A)** MA plot and **(B)** volcano plot illustrating changes to the transcriptome occurring from exposure to the wildfire-relevant exposure condition of flaming pine needles, identified through this training module. Here, each individual dot represents a gene that was queried for *via* transcriptome technologies, color-coded according to level of significance (multiple test corrected *p*-values) in association with exposure vs. control conditions within the mouse lung. Grey dots indicate genes that were not significant (*p* > 0.10), and colored dots indicate genes that were significant (*p* < 0.10). Dots of significant genes were further colored according to fold change (ratios of average exposed/unexposed samples), with red indicating positive fold change values (i.e., exposure-associated increased expression) and blue indicating negative fold change values (i.e., exposure-associated decreased expression) for the MA plot. For the volcano plot, colors indicate different filters that were implemented to identify levels of gene expression changes. Expression levels, fold change, and *p*-values are used to visualize the distribution of these statistical results produced from this example -omics analysis.

This training module specifically guides users through the loading, viewing, and formatting of the example transcriptomics datasets and associated metadata. Methods to carry out QA/QC of the transcriptomics data are then detailed, including background filtering, sample filtering, and identification of potential sample outliers. Data are adjusted for potential sources of heterogeneity, including mixed cell population distributions that are commonly present when analyzing bulk tissue samples. Statistical models are then designed and implemented to identify genes that were significantly differentially expressed by the evaluated biomass burn scenarios, as enabled through the commonly implemented DESeq2 statistical pipeline (Love et al., 2014). We find that exposure to both flaming and smoldering of pine needles caused substantial disruptions in gene expression profiles. LPS serves as a positive control for inflammation and produced the greatest transcriptomic response. Gene expression alterations are then summarized *via* visualizations using MA and volcano plots (Figure 6). Resulting lists of differentially expressed genes are lastly evaluated in the context of systems biology, through pathway enrichment analysis based off relationships to KEGG pathways (KEGG, 2021) using gene set analysis enabled through the PIANO package (Varemo et al., 2013). We find that pathways involved in cardiopulmonary function, carcinogenesis, and hormone signaling were altered in response to these wildfire-relevant exposure scenarios. Trainees are lastly pointed to additional resources, including further information on -omics and systems biology, as well as additional research examples that have evaluated -omic alterations occurring in relation to the environment and involved in disease (Smeester et al., 2011; Lu et al., 2014; Rager et al., 2016; Chappell and Rager, 2017; Balik-Meisner et al., 2018; Chappell et al., 2019; Manuck et al., 2021b; Chang et al., 2021). The importance of this module lies in the training of systems biology concepts and analysis of -omics data, including RNA sequencing data, which are becoming increasingly standard molecular endpoints used in the evaluation of exposure-induced biological responses and disease etiologies.

#### 3.2.5 Toxicokinetic Modeling

The objective of this module is to provide an overview of the basics of toxicokinetic (TK) modeling and how this type of modeling can be used in the high-throughput setting for environmental health research applications. TK modeling refers to the evaluation of the uptake and disposition of a chemical in the body. In this activity, the capabilities of the high-throughput TK modeling package, “httk,” are demonstrated on a suite of environmentally relevant chemicals. The httk R package implements high-throughput TK modeling, including a generic physiologically based toxicokinetic model, as well as chemical-specific parameters needed to solve the model for hundreds of chemicals (Pearce et al., 2017). Several questions are posed through this module, including:

1) What is the maximum concentration of bisphenol-A estimated to occur in human plasma, after one exposure dose of 1 mg/kg/day?
2) What is the estimated range of benzo(a)pyrene concentrations in plasma that can occur in a human population, assuming single doses of 1 mg/kg/day and steady-state conditions?
3) How many chemicals have available AC50 values to evaluate in the current ToxCast/Tox21 high-throughput screening database?
4) Based on httk modeling estimates, are chemicals with higher bioactivity exposure ratios always less toxic than chemicals with lower bioactivity exposure ratios?
5) How are chemical risk prioritization results different when using only toxicity information vs. only exposure information vs. bioactivity exposure ratios?

**FIGURE 7:**
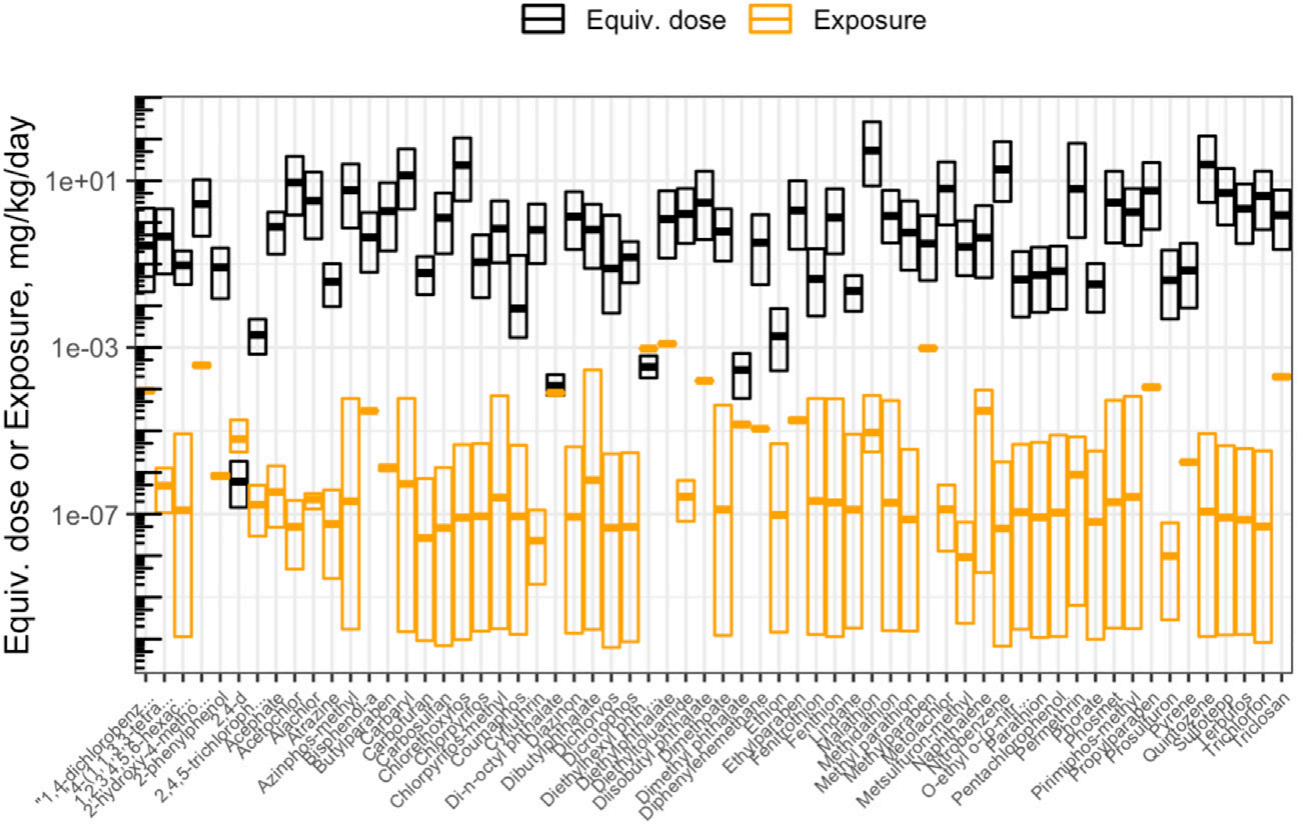
Chemicals that were analyzed using high-throughput toxicokinetic (httk) modeling in this training module. Shown here is an example visualization produced in this module illustrating how doses estimated to cause toxicity (“Equiv. dose”), that were produced through httk, compare against doses estimated as human exposures (“Exposure”). Chemicals are ranked according to bioactivity exposure ratios (BERs), indicating high potential risk (left) to low potential risk (right) to human health.

This module specifically guides trainees through a general introduction to TK, TK modeling, and the types of TK modeling that can be employed to understand how chemicals travel throughout the body. The model provides scripted examples of TK modeling, starting with the estimation of plasma concentrations over time for a human exposed to bisphenol-A. Then, population variability is considered using information from CDC National Health and Nutrition Examination Survey (NHANES) to inform a distribution of possible plasma concentrations resulting from daily exposure to benzo(a)pyrene. Then, an example high-throughput analysis is carried out over ∼1,000 chemicals, in which population variability is captured to derive estimated quantile distributions of chemical plasma concentrations during steady-state conditions of 1 mg/kg/day exposures. Trainees are then guided through the process of deriving administered equivalent doses that associate with concentrations eliciting toxicity derived through toxicity testing. Equivalent doses are specifically derived across ∼1,000 chemicals that are estimated to elicit toxicity in humans, based on *in vitro* data, through “reverse TK” calculations. The *in vitro* dataset used in these derivations is the ToxCast high-throughput screening program. ToxCast activity concentrations that elicit 50% maximal bioactivity (AC_50_) are uploaded and organized as inputs, and the 10th percentile ToxCast AC_50_ is calculated for each chemical and carried forward in the analysis as concentration estimates for potency. Bioactivity exposure ratios (BERs) are then calculated to place findings into the context of risk assessment. Here, previously generated exposure estimates that have been inferred from CDC NHANES urinary biomonitoring data are used as estimates of chemical exposures. These hazard and exposure estimates are then visualized (Figure 7). The final BERs are calculated as the ratio of the lower-end hazard equivalent dose (for the most-sensitive 5% of the population) divided by the upper-end estimated exposure (here, the upper bound on the inferred population median exposure). The importance of these BERs in chemical prioritization efforts are lastly discussed in relation to environmental health research and corresponding government regulatory decisions. Trainees are provided additional resources and cases studies that have incorporated TK/httk to address environmental health issues (Wambaugh et al., 2015; Ring et al., 2017; Klaren et al., 2019; Breen et al., 2021; Ring et al., 2021). The importance of this module is that how chemicals travel throughout the body and elicit different toxicities based upon target organs significantly depends upon toxicokinetics, and being able to model these relationships is therefore critical towards understanding chemical-induced impacts throughout the body.

#### 3.2.6 Read-Across Toxicity Predictions

The objective of this module is to provide an overview of read-across methods to computationally predict chemical toxicity based on molecular structure information. This training module represents a timely topic, paralleling increased impetus for reducing reliance upon animal testing (NAS, 2007; EPA, U.S, 2021b). In this example, data are analyzed spanning approximately 7,000 chemicals that have known structure and acute toxicity data. The specific acute toxicity endpoint that is analyzed is LD_50_, reflecting the dose required to cause lethality in 50% of animals, collected through historical animal testing. These data have been previously summarized and analyzed (Helman et al., 2019a). In this activity, we aimed to estimate an LD_50_ value for an example target chemical of interest that is commonly used in the production of industrial compounds, 1-chloro-4-nitrobenzene. To achieve this aim, we explore ways in which we can search for structurally similar chemicals that have LD_50_ data already available. Data on these structurally similar chemicals, termed “analogues,” are then used to predict acute toxicity for the target chemical. The following questions are addressed throughout this module:

1) How many chemicals with acute toxicity data are structurally similar to 1-chloro-4-nitrobenzene?
2) What is the predicted LD_50_ for 1-chloro-4-nitrobenzene, derived from read-across?
3) How different is the predicted vs. experimentally observed LD_50_ for 1-chloro-4-nitrobenzene?

**FIGURE 8:**
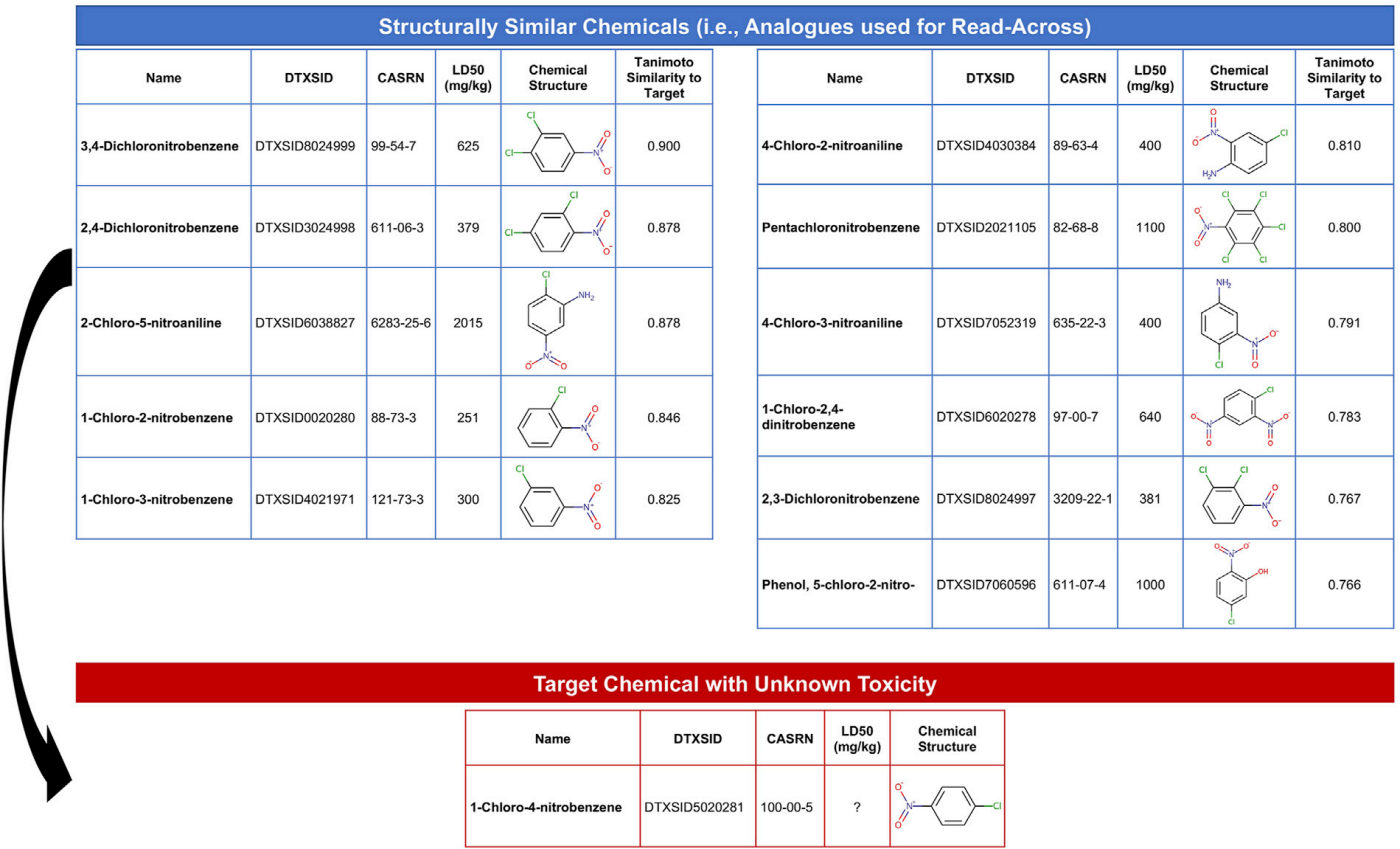
Overall schematic summarizing the steps employed in this example read-across analysis to predict chemical toxicity. This training module guides trainees through the generation of chemical structure fingerprint data and use of these data to identify analogues that can be used to predict toxicity for chemicals lacking data. This example uses chemicals with acute toxicity data (LD_50_ values) to predict an example target chemical’s acute toxicity that is structurally similar.

This module specifically guides trainees through the loading of required packages and example data, and then carries out an example read-across analysis specifically using the generalized read-across method (GenRA) (Shah et al., 2016). Trainees are guided through viewing the distribution of LD_50_ values across all evaluated chemicals. Steps are then detailed to convert SMILES nomenclature into computed molecular fingerprint data. Using these molecular fingerprint data, the degree to which each chemical is structurally similar to another chemical is evaluated based on the Tanimoto similarity index. This structural similarity analysis yields an overall similarity matrix, containing all possible pairwise similarity values. Data are then filtered to focus on chemicals with Tanimoto similarity values >0.75 to the target chemical, 1-chloro-4-nitrobenzene, resulting in a list of 11 chemical analogues that could then be used to predict toxicity for the target chemical (Figure 8). Finally, generalized read-across was carried out by calculating a similarity-weighted activity score (Shah et al., 2016), using information from the 11 analogues to predict a LD_50_ for 1-chloro-4-nitrobenzene. This *in silico* prediction was then compared to the experimentally observed LD_50_ value for this chemical, which were very similar, highlighting the utility of read-across models to inform and predict toxicity for chemicals lacking data. The importance of this module is that predicting chemical-induced toxicity using entirely *in silico* approaches represents a highly efficient skillset that scientists can leverage to better understand chemical-toxicity relationships and predict which chemicals may induce harm to public health.

### 3.3 Environmental Health Database Mining

This series of TAME Toolkit training modules covers introductory-level approaches to mining and analyzing data that can be accessed through publicly available environmental health databases. Modules span topics of mining the Comparative Toxicogenomics Database (CTD) (module 3.1), Gene Expression Omnibus (GEO) (3.2), and database integration across Air Quality, Mortality, and Environmental Justice data (3.3). These training modules also include applications-based environmental health questions and are described below.

#### 3.3.1 Comparative Toxicogenomics Database

The objective of this module is to provide an exercise on organizing and analyzing chemical-gene lists aggregated through the Comparative Toxicogenomics Database (CTD) (CTD, 2021; Davis et al., 2021). Data were specifically pulled for published chemical-gene relationships mapping to the example environmental contaminant, arsenic. The following environmental health questions were addressed through this training module:

1) Which genes show altered expression in response to arsenic exposure?
2) Of the genes showing altered expression, which may be under epigenetic control?

**FIGURE 9:**
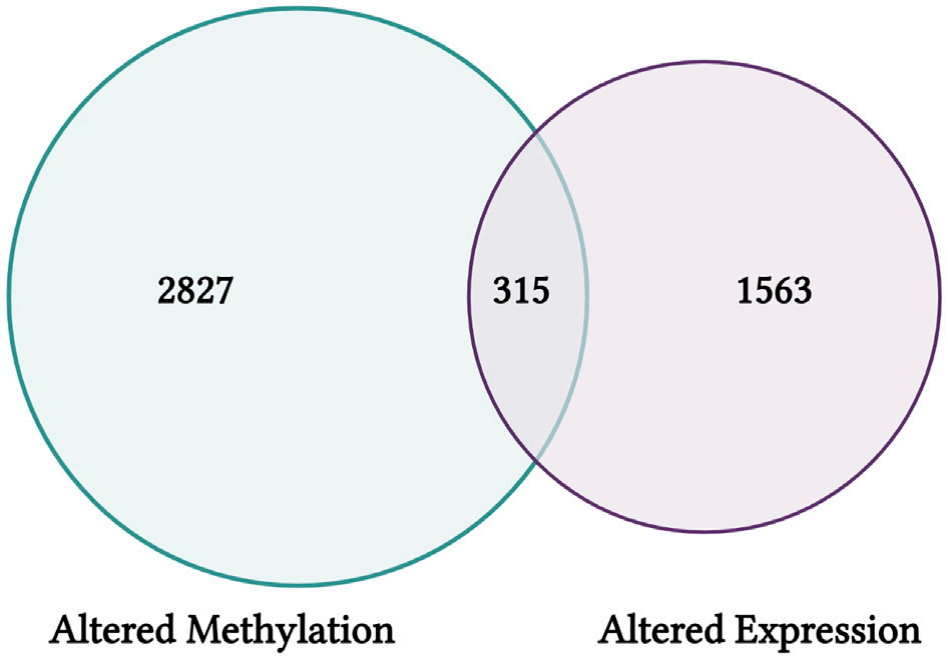
CTD findings from this training module highlight genes that have shown differential CpG methylation (left) and differential expression (right) in association with arsenic exposure. Results are visualized here using an example Venn diagram, highlighting a group of 315 genes with altered expression that may be influenced *via* epigenetic regulators through CpG methylation alterations.

This module specifically guides trainees through steps used to query CTD, including the specific selections used in this training dataset to organize chemical-gene interaction data for arsenic. These data are then uploaded into the training module R environment and used as an example for trainees to learn how to view file content and overall dimensions. Then data are filtered to include chemical-gene interactions that map specifically to changes in expression levels, yielding a list of genes that show arsenic-associated expression changes compiled from published literature. Data are additionally filtered using a different approach to yield genes that also show arsenic-associated gene methylation changes. These gene lists are then compared to result in the final elucidation of arsenic-altered genes that have published evidence for epigenetic modifications. Resulting genes represent critical mediators of inflammation and oxidative stress, among other important cellular processes. A visualization of these gene list comparison results is also scripted for using Venn diagram illustrations (Figure 9). Trainees are then provided additional resources, including reference to additional case examples that leveraged data from CTD to identify new mechanisms of environmental exposure-induced disease (Ahir et al., 2013), fill gaps on data poor chemicals to elucidate environmental influences on disease pathways (Kosnik et al., 2019), and derive new chemical risk values for prioritizing links between environmental factors, genetic variants, and human diseases (Kosnik and Reif, 2019). Together, this training module serves as an applications-based example to learn basic data manipulation, filtering, and organization steps in R, while highlighting the utility of CTD to identify novel genomic/epigenomic relationships to environmental exposures. The importance of this module is that analyzing data within CTD represents a powerful skillset within the environmental health field, which can be leveraged to improve the understanding of environmental influences on disease outcomes.

#### 3.3.2 Gene Expression Omnibus

The objective of this module is to provide an overview of pulling, organizing, visualizing, and analyzing -omics data from the GEO database (NCBI, 2021). Data were specifically pulled from an example GEO dataset (accession number GSE42394) representing gene expression data originally used in a publication evaluating the genomic effects of formaldehyde inhalation exposure in the rat (Rager et al., 2014b). The following environmental health questions were addressed through this training module:

1) What kind of molecular identifiers are commonly used in microarray-based -omics technologies?
2) How can we convert platform-specific molecular identifiers used in -omics study designs to gene-level information?
3) Why do we often scale gene expression signatures prior to heat map visualizations?
4) What genes are altered in expression by formaldehyde inhalation exposure?
5) What are the potential biological consequences of these gene-level perturbations?

**FIGURE 10:**
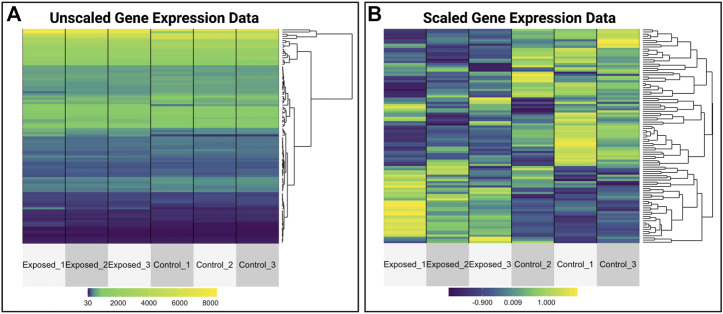
Heat map visualizations of gene expression data that are produced as examples within the GEO training module. This training module guides trainees through visualizing normalized gene expression data and highlights the differences between plotting **(A)** unscaled versus **(B)** scaled values. This example shows the utility of scaling data prior to visualizations, allowing for improved visualizations of patterns between samples.

This module specifically guides trainees through the loading of required packages and data, including the manual upload of GEO data as well as the automated upload of data leveraging the GEO query package. Data are then further organized for downstream analyses. Trainees are then provided an overview of the types of molecular identifiers used in this example dataset, originally centered around microarray-based probeset identifiers. To increase interpretability of analysis findings, methods to merge platform-specific identifiers with gene-level annotation information are carried out. Example visualizations are then produced, including boxplots to evaluate the overall distribution of expression data across samples, as well as heat map visualizations that compare unscaled versus scaled gene expression values to emphasize the utility of scaled values for improved visualization of patterns between samples (Figure 10). Statistical analyses are then included to identify which genes are the most significantly altered in expression upon exposure to formaldehyde. The gene identified with the most significantly increased expression in the rat nose is olfactory receptor 633 (*Olr633*), demonstrating that formaldehyde inhalation exposure induced olfactory-related signaling. Together, this training module serves as an important example on how scientists can efficiently leverage existing genome-wide datasets to address new environmental health questions. Trainees are also pointed to previous publications applying these methods to existing GEO datasets that address additional environmental health questions (Rager and Fry, 2012; Rager et al., 2019). The importance of this module is that online -omics databases, such as GEO, represent robust resources that can be mined to better understand mechanisms of disease and biological responses to insults, and becoming familiar with such resources will expand data reusability and interpretation in future environmental health studies.2

#### 3.3.3 Database Integration: Air Quality, Mortality, and Environmental Justice Data

The objective of this module is to provide an example analysis based on the integration of data across multiple environmental health databases. Specifically, air quality monitoring data from the U.S. EPA’s Air Quality System (AQS) (EPA, U.S, 2021a) were analyzed, focusing on the 2016 EPA Monitoring Data Annual Average database. These data included average measures of particles ≤2.5 μm in diameter (PM_2.5_), nitrogen dioxide (NO_2_), and sulfur dioxide (SO_2_). Health outcome data were also analyzed, specifically from the Center for Disease Control (CDC) Wide-ranging ONline Data for Epidemiologic Research (WONDER) database (CDC, 2021). These data included the 2016 all-cause mortality rates. Population-level variables were additionally analyzed, including race, and included in the statistical modeling as well as the evaluation of population-level information that can be used to examine Environmental Justice issues. All data were pulled and summarized at the county-level across the entire U.S. for the year 2016 (Remington et al., 2015; UWPHI, 2021). The following environmental health questions were addressed through this training module:

1) What areas of the U.S. are most heavily monitored for air quality?
2) Is there an association between long-term, ambient PM2.5 concentrations and mortality at the county level?
3) What is the difference when running crude statistical models vs. statistical models that adjust for potential confounding, when evaluating the relationship between PM_2.5_ and mortality?
4) Do observed associations differ when comparing between counties with a higher vs. lower percentage of African-Americans which can indicate Environmental Justice concerns?

**FIGURE 11:**
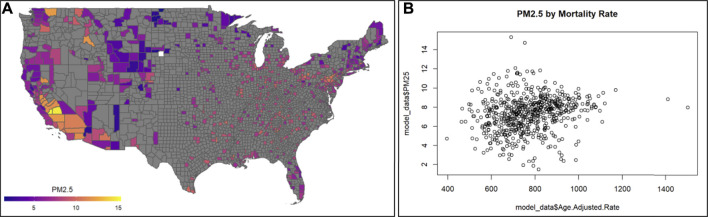
Example visualizations of **(A)** air quality data, highlighting the 2016 annual PM_2.5_ concentrations across U.S. counties, and **(B)** potential relationships against mortality rates. Code that supports the generation of these visualizations and example statistics evaluating trends between air pollution and mortality rates, and analyses including potential confounders, is described within this training module.

This module specifically guides trainees through an explanation of how the data were downloaded and organized, and then details the loading of required packages and datasets. Then, this module provides code for visualizing county-level air pollution measures obtained through U.S. EPA monitoring stations throughout the U.S. Air pollution measures include PM_2.5_, NO_2_, and SO_2_, and are visualized here as the yearly average (Figure 11A). Air pollution concentrations are then evaluated for potential relationship to the health outcome, mortality (Figure 11B). Specifically, age adjusted mortality rates are organized and associated with PM_2.5_ concentrations through linear regression modeling. Crude (univariate) statistical models are first provided that do not take into account the influence of potential confounders. Then, statistical models are used that adjust for potential county-level confounders, including adult smoking, obesity, food environment indicators, physical activity, employment status, rural vs. urban living percentages, sex, ethnicity, and race. Results from these models point to the preliminary finding that PM_2.5_ is associated with elevated county-level mortality rates. Previous studies have shown that minority populations reside closer to air pollution sources and as a result are exposed to poorer air quality. This is also seen in these data, with measured air quality differing by percent African-American race in each county. Race is then evaluated further in this analysis as a potential differentiating factor in the models. Here, data distributions are pulled for counties with the highest percentage of African-Americans (top 25%) as well as those with the lowest percentage of African-Americans (bottom 25%). Models associating PM_2.5_ with all-cause mortality rates are then re-run in these groups and the PM_2.5_-mortality associations are compared. Counties with the highest percentages of African-American race had a significant association with mortality, with magnitudes substantially greater than counties with the lowest percentages of African-American race. This result corresponds with known Environmental Justice concerns, and demonstrates how even a cross-sectional, ecological analysis can highlight differences in environmental health risks. The importance of this module is that it demonstrates ways to integrate disparate health and environmental exposure databases in order to study key questions in environmental public health, including examinations of important Environmental Justice issues.

#### 3.3.4 Additional Resources

A final module is included that lists additional resources to aid in the continued training of users on data management and analysis strategies. We specifically include online websites and other training resources that we have found to be useful towards programming and data analysis approaches. These resources are sorted into the following four categories: 1) R programming resources; 2) R packages resources; 3) community discussions on R and R packages; 4) R interfaces; and 5) data science and statistical analysis resources.

## 4 Discussion and Conclusion

Together, this TAME Toolkit aims to serve as a helpful resource to promote trainee-driven data generation, management, and analysis methods to address the growing demands of 21st century environmental health concerns. Training modules are provided to serve as timely data analysis examples, all describing methods used to extract meaningful results to inform environmental health research applications. R was selected as the example coding platform, leveraging R Markdown and Bookdown formatting; though we recognize that additional training across other computing platforms could expand trainee data analysis skills and capabilities. The training modules are not designed as an exhaustive list of all resources and techniques available to analyze data relevant to environmental health. Rather, these modules highlight examples of methods and databases that can be leveraged in this research field, such that trainees can effectively navigate their way through each training lesson and translate methods learned to future questions. Modules were designed as a starting point for a data analysis technique, where additional resources are provided for further learning opportunities and technical guidance. The content within each module was selected to highlight important methods that can enhance environmental health studies, and thus, these modules collectively provide a cohesive set of skills that participants can leverage within their current research studies, real-world applications, and/or future job marketability, depending on their career stage. In conclusion, this resource serves as a unique training opportunity for future data analysts to learn timely data science and analysis methodologies in an applications-driven manner relevant to environment health research.

## Data Availability

The original contributions presented in the study are included in the article and online through the TAME Toolkit, available at: https://uncsrp.github.io/Data-Analysis-Training-Modules/, with underlying code and datasets available in the parent UNC-SRP GitHub website (https://github.com/UNCSRP).
